# Targeting duplex DNA with the reversible reactivity of quinone methides

**DOI:** 10.1038/sigtrans.2016.9

**Published:** 2016-06-24

**Authors:** Chengyun Huang, Yang Liu, Steven E Rokita

**Affiliations:** 1 Department of Chemistry and Biochemistry, University of Maryland, College Park, MD, USA

## Abstract

DNA alkylation and crosslinking remains a common and effective strategy for anticancer chemotherapy despite its infamous lack of specificity. Coupling a reactive group to a sequence-directing component has the potential to enhance target selectivity but may suffer from premature degradation or the need for an external signal for activation. Alternatively, quinone methide conjugates may be employed if they form covalent but reversible adducts with their sequence directing component. The resulting self-adducts transfer their quinone methide to a chosen target without an external signal and avoid off-target reactions by alternative intramolecular self-trapping. Efficient transfer is shown to depend on the nature of the quinone methide and the sequence-directing ligand in applications involving alkylation of duplex DNA through a triplex recognition motif. Success required an electron-rich derivative that enhanced the stability of the transient quinone methide intermediate and a polypyrimidine strand of DNA to associate with its cognate polypurine/polypyrimidine target. Related quinone methide conjugates with peptide nucleic acids were capable of quinone methide transfer from their initial precursor but not from their corresponding self-adduct. The active peptide nucleic acid derivatives were highly selective for their complementary target.

## Introduction

**Figure 1:**
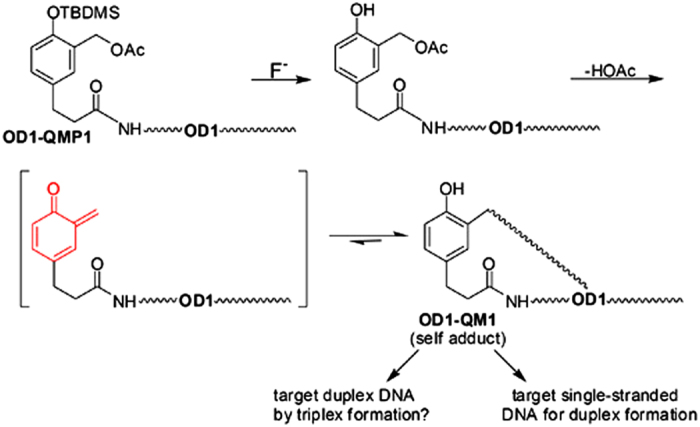
Design of a reversible quinone methide (QM) conjugate for targeting nucleic acids. **OD1**-QMP1 represents the conjugate between oligonucleotide 1 and the quinone methide precursor 1 illustrated in Figure 4. TBDMS is used to abbreviate *tert*-butyldimethylsilyl groups.

Covalent chemistry is not often considered in the design of pharmaceutical compounds despite its success in anti-cancer treatments and periodic resurgence in drug development.^1^ Off-target reactions are often difficult to control and cause premature consumption of the active species. These same side reactions can also be responsible for the toxic effects of compounds reacting covalently. Such problems may be mitigated by conjugating the reactive center to a site-directing component but then the kinetics of reaction must be balanced with the kinetics of distribution and ultimately target association. Alternatively, prodrug conjugates can be constructed for delivery in a protected form followed by selective activation that may be designed to commence after binding to the target. Quinone methides (QMs) and related electrophiles can be generated transiently through a range of strategies to alkylate various targets and these intermediates have become central to numerous applications in chemistry and biology.^2–4^ For example, QM formation can be initiated by reduction,^5^ oxidation^6–9^ and photo- and thermal excitation.^10,11^ Certain QMs may also react reversibly with nucleophiles to avoid irreversible consumption and unintended alkylation. Such reversibility has allowed for exchange and migration of DNA crosslinking in processes that are driven by the thermodynamics of the products rather than the initial kinetics of reaction.^12,13^ QM conjugates also provide a means of sequence-directed alkylation of single-stranded nucleic acids without need of prodrug activation.^14–16^ Initial deprotection of a latent QM can form self-adducts with their sequence-directing component as illustrated in Figure 1. These self-adducts (**OD1**-QM1) spontaneously and reversibly regenerate their QM intermediates and remain unaffected by competing nucleophiles such as thiols due to the high efficiency of intramolecular reaction.^12,14^ QM transfer to date has only been observed from oligonucleotide self-adducts to their single-stranded complements.

**Figure 2:**
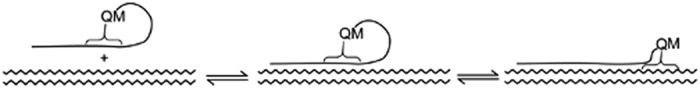
Possible equilibria controlling target-specific alkylation. QM represents a quinone methide adduct formed with the DNA.

Single-stranded targets of oligonucleotide-QM self-adducts are useful in studying the potential of reversible reaction in sequence-directed reactions *in vitro* but do not offer compelling applications *in vivo* due to the numerous and well-established alternatives for disrupting gene translation.^17,18^ In contrast, delivery of a QM self-adduct to duplex DNA may provide a number of unique opportunities since few options exist for controlled reaction of duplex DNA. A number of sequence-specific ligands have been developed for manipulating gene expression,^19–22^ but only covalent chemistry will suppress dissociation during the energy-dependent unwinding and denaturation of DNA during replication. A variety of conjugates have previously been generated to recognize the major groove of polypurine/polypyrimidine regions in duplex DNA and deliver an appendage for covalent reaction. Off-target reaction can be suppressed by use of appendages with latent chemistry. Reaction can be controlled on demand with signals ranging from reduction,^23^ to oxidation^24,25^ and photoexcitation.^26^ Each condition has certain advantages and disadvantages. Ultimately, the most broadly applicable method will likely include an induction method as simple and universal as target binding.^27,28^ Self-adducts of QMs have the potential to satisfy this desire as efficient transfer of QMs has only been detected within target complexes and, if alternative reaction occurs, their reversibility will still support final accumulation at the thermodynamically favored site.^12^ The utility of oligomer-QM self-adducts in triplex recognition and reaction was not directly apparent from earlier models that relied on Watson–Crick base pairing. Energies associated with binding of a third strand of DNA is not as large as that for the initial formation of the duplex. The structural perturbations caused by the self-adduct were also a concern as these could suppress triplex formation (Figure 2). Similarly, the relative thermodynamics of QM transfer to the duplex target relative to reformation of the triplex-forming self-adduct were not obvious from prior study (Figure 2). Triplex-forming self-adducts have now been developed as described below to deliver a QM in a selective manner. Success very much depends on the sequence of the target and nature of the self-adduct.

## Materials and methods

### General materials

DNA was purchased from Integrated DNA Technologies (Coralville, IA, USA). **PNA1** was prepared according to the literature.^16^ The *N*-hydroxysuccinimide esters of the QM precursors NHS-QMP1 and NHS-QMP2 (Figure 4) were prepared as described previously.^14,29^ Aqueous solutions were prepared from deionized water with a resistivity of 18.0 MΩ. T4 polynucleotide kinase (PNK) was obtained from New England Biolabs (Ipswich, MA, USA). γ-[^32^P]-ATP was purchased from Perkin-Elmer (Waltham, MA, USA). Micro bio-spin columns with bio-gel P-6 was purchased from Bio-Rad Laboratories (Hercules, CA, USA).

### General procedures

Target DNA was radiolabeled at the 5ʹ-terminus with γ-[^32^P]-ATP and PNK using standard procedures recommended by the manufacturer. Duplex DNA was annealed by heating to 90 °C for 3 min followed by cooling to ambient temperature over more than 3 h under the pH and ionic conditions used for subsequent reaction. The DNA and peptide nucleic acid analog (PNA) conjugates of QMP1 and QMP2 were prepared by combining equal volumes of the appropriate oligomer in 3-(*N*-morpholino)propanesulfonate buffer (MOPS, 250 mM, pH 7.5) and NHS ester in CH_3_CN/DMF (2:1) for 24 h at ambient temperature as described in the literature.^16,29^ The desired products were purified by reverse-phase (C-18) HPLC and identified by MALDI-TOF mass spectrometry (Supplementary Figures S1 and S2). Preparation of the oligonucleotide self-adducts (**OD1**-QM1 and **OD2**-QM2) followed literature procedures^29^ and was confirmed with MALDI-TOF mass spectrometry (Supplementary Figures S1 and S2). The PNA self-adduct **PNA1**-QM2 was generated equivalently. **PNA1**-QMP2 (20 μM) in 2-(*N*-morpholino)ethanesulfonate (MES) (25 mM pH 7.0) was treated with KF (670 mM) under ambient conditions for 24 h. The resulting self-adduct was purified by reverse-phase (C-18) HPLC using a linear gradient of 10–55% aqueous acetonitrile with 0.1% trifluoroacetic acid. MALDI-TOF mass spectrometry confirmed successful generation of this self-adduct as well (Supplementary Figures S2).

### Alkylation of target DNA

Individual reaction conditions are described in the legends of each figure. When MgCl_2_ was present in the reaction mixtures, samples were desalted with Micro Bio-Spin columns before analysis. All samples were combined with equivalent volumes of a loading solution (0.05% xylene cyanol and 0.05% bromphenol blue in formamide) and separated by denaturing polyacrylamide (20%) gel electrophoresis. Target alkylation was quantified by phosphoimagery and the yields were calculated as a percent of total signal.

## Results and discussion

### Alkylation of duplex DNA with a triplex-forming conjugate alternatively containing a quinone methide precursor (QMP1) and a QM self-adduct

**Figure 3:**
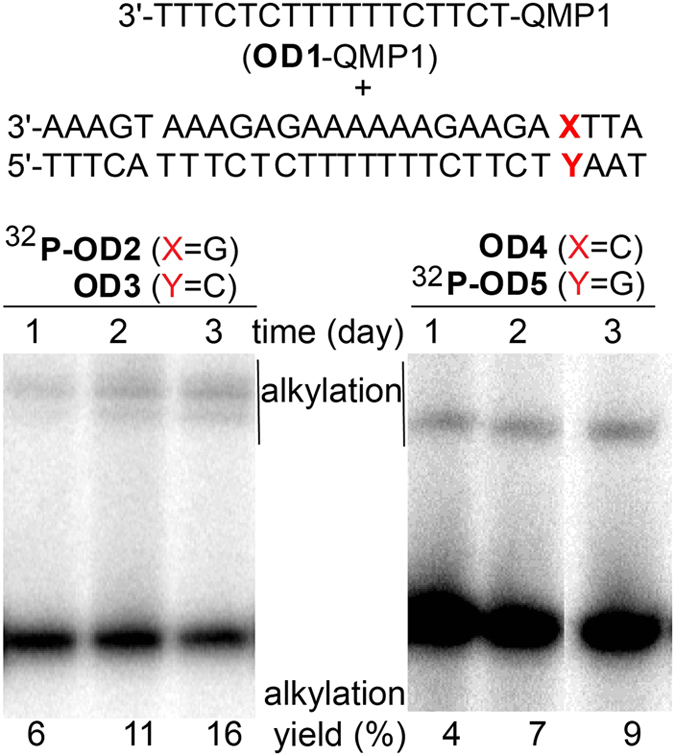
Alkylation of duplex DNA by a triplex-forming oligonucleotide conjugate. **OD1**-QMP1 (10.0 μM) was incubated with the indicated duplex (50 nM) in NaCl (150 mM), MgCl_2_ (2.5 mM) and MES (20 mM pH 5) for 1–3 days before analysis by denaturing polyacrylamide (20%) gel electrophoresis. The yield of alkylation (%) was determined by phosphoimagery and presented as the fraction of total signal per lane. QMP1 represents the quinone methide precursor illustrated in Figure 4.

The chemical competence for alkylation of duplex DNA by a QM delivered to the major groove through triplex recognition was confirmed using a target based on a sequence within the *Hprt* gene. This sequence had previously been tested successfully with a psoralen conjugate that induced crosslinking by UV irradiation.^26^ The natural sequence contains the necessary polypurine/polypyrimidine tract and terminates with a 5'-TA-3' that supports psoralen crosslinking. In contrast, QM had demonstrated preferential reaction at G in the major groove^30^ and hence the final 5'-TA-3' was replaced with 5'-GA-3' (Figure 3). Previous studies with a different target sequence and a bifunctional QMP suggested that a G directly adjacent to the triplex site-dominated reaction and a G contiguous with the polypurine strand was alkylated more efficiently than a G contiguous with the polypyrimidine strand.^31^ This hierarchy was much more subtle for the oligonucleotide conjugate of QMP1 (**OD1**-QMP1) as indicated by the modest yields of high-molecular-weight species separated from the parent target after denaturing gel electrophoresis (Figure 3). The apparent preference for reaction at G adjacent to the polypurine strand (X=G, **OD2**) was slight compared with reaction of G adjacent to the polypyrimidine strand (Y=G, **OD5**) and even their complementary strands lacking such a G were still subject to low levels of alkylation (Supplementary Figure S3). Both C and A have the potential for alkylation by QMs and the yield of the A adduct can be significantly underestimated due to its kinetic lability.^32,33^ Regardless of these complications, our initial examination successfully demonstrated that a triplex-forming strand can position a QMP in the major groove and nucleophiles within this site are available for alkylation by the nascent QM.

The more significant challenge is to alkylate the target DNA by QM transfer from the conjugate’s self-adduct (**OD1**-QM1) rather than from its initial precursor as the former condition requires no external induction. Of course, this presumes that **OD1** can support and maintain reversible self-adducts. T remains inert to the parent QM1 substructure,^14,32^ and thus self-adduct formation necessarily relies on the remaining Cs. These residues are distributed sparsely in **OD1** but were still sufficient for capture of the QM. The self-adduct was isolated by reverse-phase (C-18) chromatography after deprotection of its precursor **OD1**-QMP1. The identity of the self-adduct was confirmed by MALD-TOF mass spectrometry (Supplementary Figure S1). Unfortunately, this self-adduct did not alkylate the target duplex (**OD2**/**OD3**) even after extended incubation (3 days). An analogous self-adduct (^**Me**^**OD1**-QM1) was consequently prepared in which the C residues were replaced with 5-MeC. This substitution stabilizes triplex formation^34^ and had the potential to compensate for the destabilization or structure deformation of a triplex assembly that might have been caused by the self-adduct. This modification of the site-directing component was still not sufficient to promote target alkylation as no high-molecular-weight derivative of the sequence (**OD2**) was detected after incubating its duplex with the modified self-adduct ^**Me**^**OD1**-QM1 (Supplementary Figure S4).

**Figure 4:**
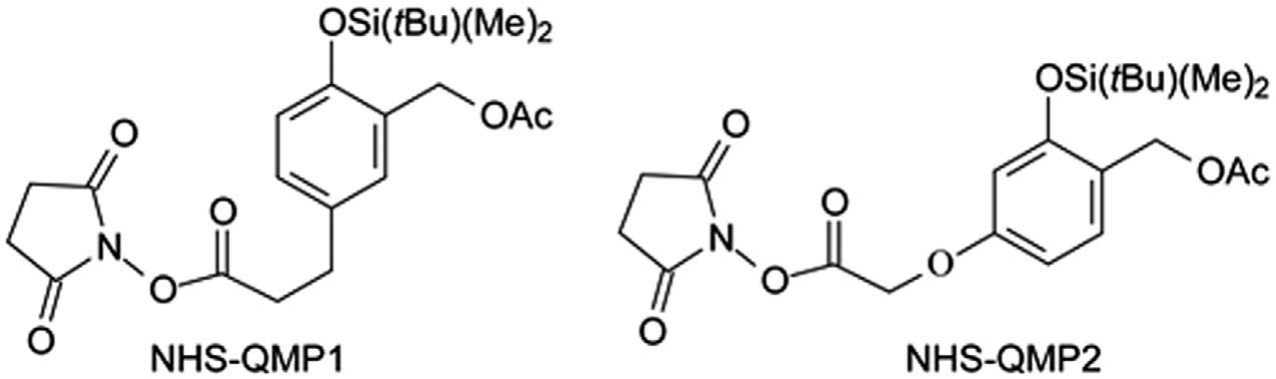
*N*-hydroxysuccinimide esters (NHS) used to make conjugates of QMP1 and QMP2.

Another strategy to enhance an association between a QM conjugate and its target DNA is to replace the sequence-directing oligonucleotide with a PNA. This alternative retains the same nucleobases of DNA but replaces the phosphoribose backbone with an unnatural peptide-like backbone.^35^ PNA is resistant to proteases and its lack of charge avoids the electrostatic repulsion that suppresses triplex formation with the appropriate oligonucleotide.^36^ These same features also facilitate the intracellular delivery of PNA.^37^ On the basis of reports that C-rich PNA increases the potential to form a triplex structure with a complementary DNA,^38^ a different duplex was prepared as a model target (**OD6**/**OD7**). Its sequence represents a polypurine–polypyrimidine region within the transcription factor gene for NF-κB and contains sufficient G/C pairs for assembly with a C-rich PNA.^39^ The appropriate PNA was consequently prepared by standard solid-phase synthesis and capped on both termini with arginine (Arg) to enhance its solubility and affinity for DNA.^16^ A short linker was also included for coupling to the activated ester of QMP1 (NHS-QMP1, Figure 4). Results with this new construct were once again disappointing. No high-molecular-weight products consistent with target alkylation were evident after a 50-fold excess of **PNA1**-QMP1 was induced to form its QM in the presence of labeled **OD6** and its complement **OD7** (Supplementary Figure S5, lane 2). This negative result dissuaded further work with QMP1 and its self-adducts.

### Enhanced reactivity of an electron-rich quinone methide (QM2)

Modulation of QM reactivity provides an alternative variable that may promote the desired alkylation of duplex DNA. Both the kinetics of reversible alkylation and the stability of this electrophilic intermediate are very sensitive to substituent effects.^40–42^ Electron-donating groups stabilize QMs and promote both their initial formation and regeneration from their alkylated products. Likewise, electron-withdrawing groups suppress QM formation and its regeneration. Changes in the rate of formation and consumption also necessarily affect the lifetime of the transient intermediate. These principles were initially observed in model systems and have since been applied to self-adduct formation, sequence-directed alkylation, and crosslink mobility based on QMs.^13,29^ In these examples, a methylene group was replaced by an ether for linking the DNA ligand to the QMP. This substitution provided the necessary electron donation to the nascent QM for its rapid exchange of alkylation. In particular, electron-rich substituents *para* to the nascent *exo*-methylene group of the QM have the most dramatic effect.^40^ Thus, the activated ester of QMP2 (NHS-QMP2, Figure 4) was prepared and coupled to **PNA1** in a new attempt to alkylate the duplex **OD6**/**OD7**.

**Figure 5:**
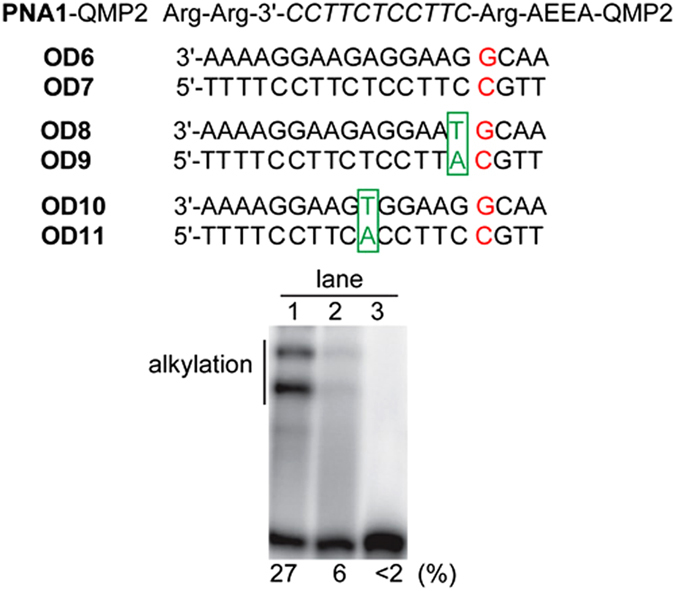
Target specificity for alkylation of duplex DNA by a PNA conjugate. 5-[^32^P]-**OD6**/**OD7** (50 nM, lane 1), 5-[^32^P]-**OD8**/**OD9** (50 nM, lane 2), and [^32^P]-**OD10**/**OD11** (50 nM, lane 3) were treated the **PNA1-**QMP2 conjugate (2.5 μM) in NaCl (50 mM), NaF (100 mM) and sodium phosphate (10 mM pH 6.0) at 37 °C for 3 days and analyzed by denaturing polyacrylamide (20%) gel electrophoresis. The yield of alkylation (%) was determined by phosphoimagery and represents the fraction of total signal per lane. The PNA residues are noted in italics and AEEA represents the 2-[(2-amino)ethoxy)]ethoxy acetyl group used as a linker.

The enhanced stability of the electron-rich QMP2 successfully supported duplex alkylation. Unmasking the QM of **PNA1**-QMP2 resulted in alkylation of its target duplex to a yield of >25% within 48 h (Supplementary Figure S6). Thus, adaptation of the QM was able to achieve what stabilization of the triplex structure could not. Of course, specificity is a key requirement for sequence-directed reaction. Target selectivity was measured by the relative reactivity of duplex sequences containing single mismatches within the recognition region of triplex assembly. **PNA1**-QMP2 was challenged with **OD8**/**OD9** containing a single G/C to T/A substitution at the triplex terminus. Yield of its alkylation was depressed by more than four-fold relative to the fully complementary **OD6**/**OD7** (Figure 5). Alternative substitution of a A/T to T/A pair internal to the triplex region completed inhibited reaction under comparable conditions (**OD10**/**OD11**, Figure 5). Thus, alkylation was both effective and selective. Its sensitivity to the target sequences suggests that triplex assembly was a prerequisite for reaction. The internal mismatch destabilizes the triplex formation to a greater extent than a terminal mismatch and the decreasing yields of alkylation reflect this same trend. A similar discrimination for a single substitution within a target was similarly observed when a PNA conjugate was used to direct alkylation to its complementary single-stranded DNA.^16^

The specificity exhibited by **PNA1**-QMP2 is sufficient to consider future development toward related conjugates designed to suppress dominant lethal mutations created by nucleotide polymorphisms. However, this will require use of the corresponding self-adduct of a QM conjugate that is capable of alkylating its target without addition of an external trigger for unmasking the QM. Accordingly, the self-adduct **PNA1**-QM2 was generated, isolated by reverse-phase chromatography and confirmed with MALDI-TOF mass spectrometry (Supplementary Figure S2). However, incubation of this species with its complementary target of **OD6**/**OD7** did not produce the desired alkylation product (Supplementary Figure 5S, lane 1). This result likely reflects a decrease in the self-adduct’s ability to associate with the target duplex relative to that of its precursor (Figure 2), as **OD6**/**OD7** already demonstrated a susceptibility to alkylation (Figure 5 and Supplementary Figure S6). Partitioning of the QM between self-alkylation and target alkylation should be independent of the QM’s history whether generated from **PNA1**-QMP2 or its self-adduct **PNA1**-QM2.

**Figure 6:**
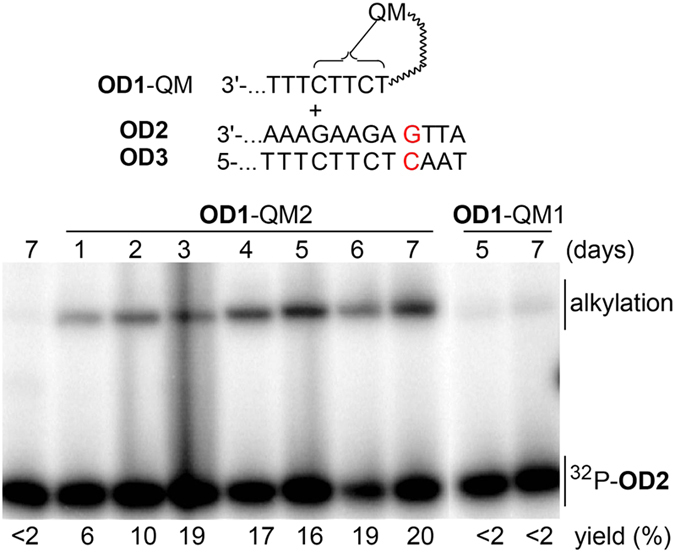
Spontaneous alkylation of duplex DNA by DNA-quinone methide self-adducts (**OD1**-QM1 and **OD1**-QM2). 5'-[^32^P]-**OD2**/**OD3** (0.5 μM) was treated with the indicated self-adducts (5 μM) in NaCl (150 mM), MgCl_2_ (2.5 mM) and MES (20 mM pH 5) for 1–7 days at ambient temperature before analysis by denaturing polyacrylamide (20%) gel electrophoresis. The yield of alkylation (%) was determined by phosphoimagery and represents the fraction of total signal per lane.

Increasing the binding affinity of the conjugate by extending the length of PNA has the potential to overcome the deficiency of the self-adduct **PNA1**-QM2. However, such an extension also increases the risk of off-target recognition and reaction as well. Instead, the success of the electron-rich QMP2 encouraged a return to the original conjugate and target system based on DNA. This would address questions on the limited yield initially observed with triplex assembly and reaction of **OD1**-QMP1 (Figure 3) and its self-adduct **OD1**-QM1 (Supplementary Figure S4). The alternate conjugate **OD1**-QMP2 was synthesized by coupling **OD1** with the activated QMP2 (Figure 4) and subsequently used to generate its self-adduct **OD1**-QM2 under standard conditions. After purification by reverse-phase HPLC, the collected material appeared by MALD-TOF to contain a mixture of the self-adduct and the water-quenched product (Supplementary Figure S1). A similar mixture was also observed with the **PNA1**-QM2 self-adduct (Supplementary Figure S2). Water addition to the QM is irreversible and thus its product does not participate in target alkylation.^14,43^ At worst, this inert derivative may act as a competitive inhibitor of the self-adduct and decrease the apparent efficiency of reaction. However, its effect was not significant as only a 10-fold excess of the mixture containing **OD1**-QM2 was necessary to achieve a maximum yield of alkylating **OD2** in a duplex with **OD3** (Supplementary Figure S7). This demonstrates the first success in alkylating a DNA duplex with a triplex-forming QM self-adduct. A time course of reaction indicated a maximum yield of ~19% within 72 h (Figure 6). This yield represents a minimum estimate for the self-adduct alone as it is applied as a mixture with the inactive water adduct. Removal of this later derivative is impractical and even futile as it will accumulate spontaneously during each reversible release of the transient QM intermediate from the self-adduct and its precursor. Overall, the goal of alkylating duplex DNA using a QM that did not require an external trigger was achieved with an electron-rich derivative. This was designed to enhance the rate of QM regeneration and extend the lifetime of the electrophilic intermediate.^13,29,40^ Perhaps this modification of the QM was necessary for it to persist long enough for a structural reorganization that could be required for effective transfer of the QM from the sequence-directing ligand to target duplex DNA.

QM and their derivatives provide a valuable platform for target-selective reaction. Their reactivity and kinetics can be modulated predictably by the use of substituent effects and their selectivity can be directed by appending an appropriate ligand. Triplex-mediated alkylation of DNA has been accomplished with both oligonucleotide and PNA conjugates. Further tuning of the reaction and site-directing components should ultimately offer a general protocol for gene-specific modification.
