# Supplementary material for: Targeting duplex DNA with the reversible reactivity of quinone methides
Source: Signal Transduct Target Ther. 2016 Jun 24;1:16009–. doi: 10.1038/sigtrans.2016.9 (PMC5407369; doi:10.1038/sigtrans.2016.9)
Supplement: Supplementary Information [file sigtrans20169-s1.pdf]

Electronic Supplemental Information (ESI)

for

Targeting Duplex DNA with the Reversible Reactivity of Quinone Methides

Chengyun Huang, Yang Liu and Steven E. Rokita

Contents:

Figure S1. MALDI-TOF characterization of DNA conjugates.

Figure S2. MALDI-TOF characterization of PNA conjugates.

Figure S3. Alkylation of a target duplex by a triplex-forming oligonucleotide conjugate.

Figure S4. Alkylation of duplex DNA by a triplex-forming oligonucleotide-QM1 self-adduct.

Figure S5. Alkylation of duplex DNA by PNA conjugates.

Figure S6. Alkylation of duplex DNA by a PNA conjugate with an activated quinone methide precursor.

Figure S7. Concentration dependence of duplex alkylation by a DNA-QM self-adduct.

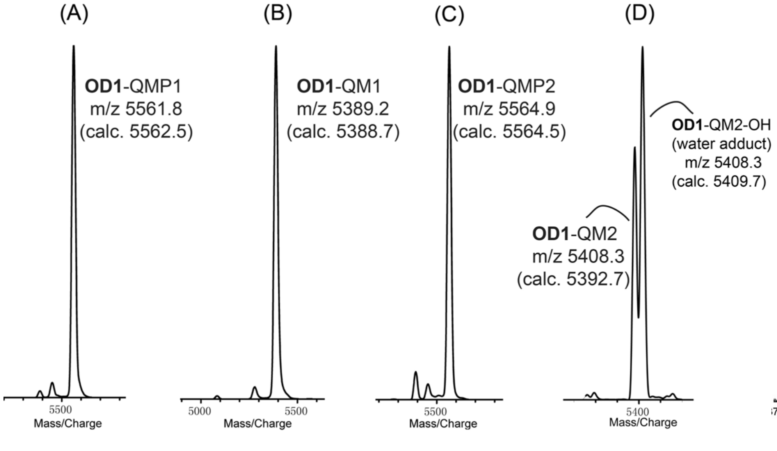

Figure S1. MALDI-TOF characterization of DNA conjugates.

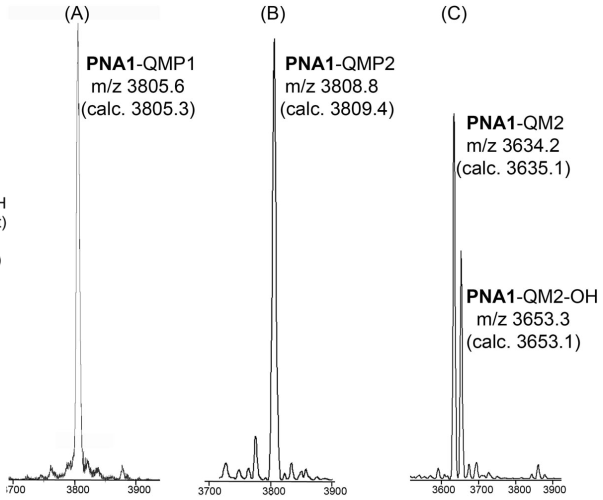

Figure S2. MALDI-TOF characterization of PNA conjugates.

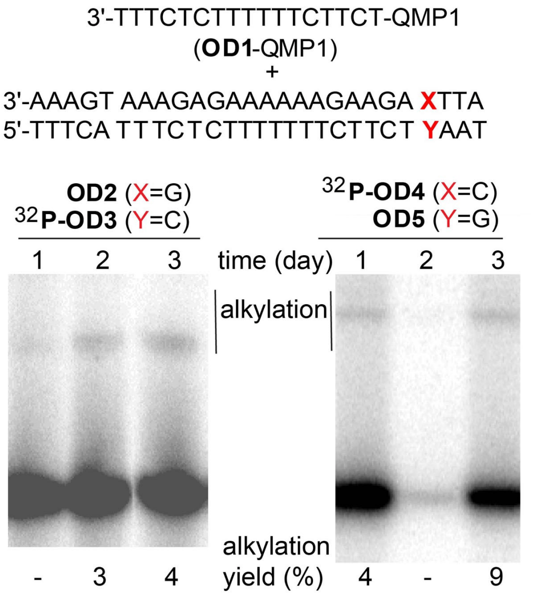

Figure S3. Alkylation of a target duplex by a triplex-forming oligonucleotide conjugate. **OD1**-QMP1 (10.0 μM) was incubated with the indicated duplex sequence (50 nM) in NaCl (150 mM), $MgCl_2$ (2.5 mM) and MES (20 mM pH 5) for 1 - 3 days before analysis by denaturing polyacrylamide (20%) gel electrophoresis. The yield of alkylation (%) was determined by phosphoimagery and represents the fraction of total signal per lane.

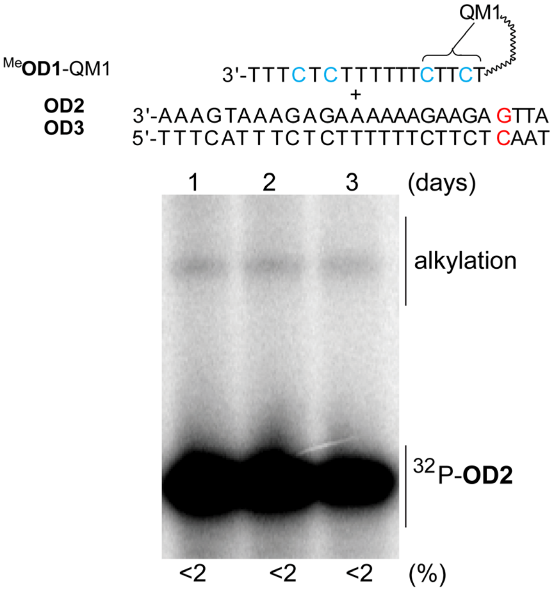

Figure S4. Alkylation of duplex DNA by a triplex-forming oligonucleotide-QM1 self- adduct. The self-adduct **OD1**-QM1 (5.0 μM) was incubated with the indicated duplex sequence (50 nM) in NaCl (150 mM), $MgCl_2$ (2.5 mM) and MES (20 mM pH 5) for 1 - 3 days before analysis by denaturing polyacrylamide (20%) gel electrophoresis. 5-Me-dC is noted in blue. The yield of alkylation (%) was determined by phosphoimagery and represents the fraction of total signal per lane.

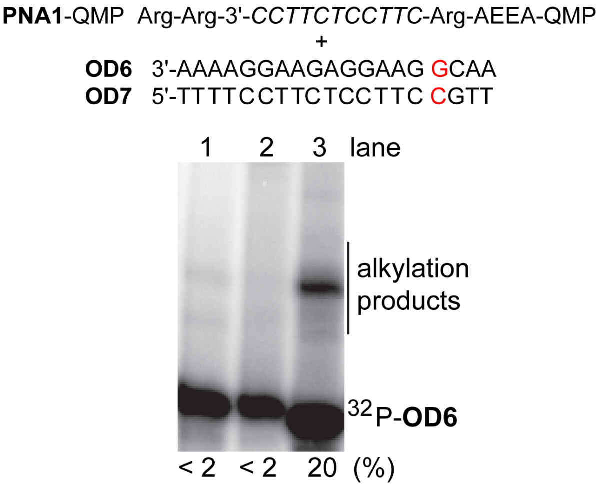

Figure S5. Alkylation of duplex DNA by PNA conjugates. The duplex **OD6**/**OD7** (50 nM) was treated alternatively with the **PNA1**-QM2 self-adduct (2.5 μM) (lane 1), the **PNA1**-QMP1 conjugate (2.5 μM) (lane 2) in NaCl (50 mM), NaF (100 mM) and sodium phosphate (10 mM pH 6). The single-stranded **OD6** (2.2 μM) was also treated under equivalent conditions with the **PNA1**-QMP2 conjugate (2.5 μM) (lane 3). Samples were incubated at 37°C for 4 days and then directly analyzed by denaturing polyacrylamide (20%) gel electrophoresis. The yield of alkylation (%) was determined by phosphoimagery and represents the fraction of total signal per lane. The PNA residues are noted in italics and AEEA represents the 2-[(2-amino)ethoxy)]ethoxy acetyl group used as a linker.

Figure S6. Alkylation of duplex DNA by a PNA conjugate with an activated quinone methide precursor. The duplex **OD6**/**OD7** (50 nM) was treated with **PNA1**-QMP2 (2.5 μM) in NaCl (50 mM), NaF (100 mM) and sodium phosphate (10 mM pH 6) for the indicated time at 37°C prior to analysis by denaturing polyacrylamide (20%) gel electrophoresis. The yield of alkylation (%) was determined by phosphoimagery and represents the fraction of total signal per lane. The PNA residues are noted in italics.

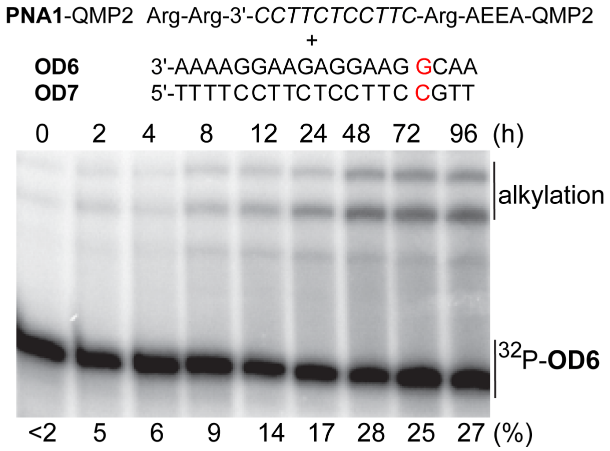

(A)

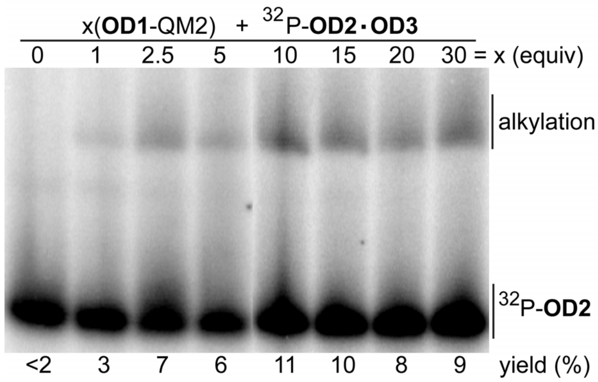

(B)

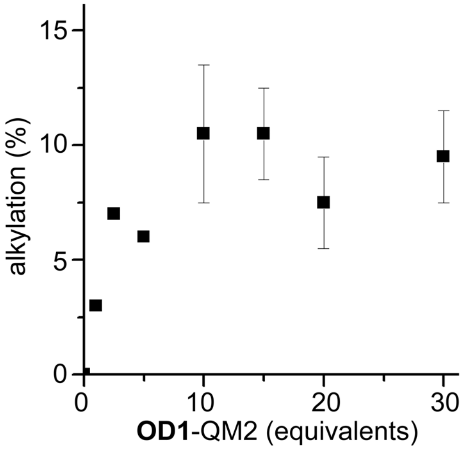

Figure S7. Concentration dependence of duplex alkylation by a DNA-QM self-adduct. (A) [^{32}P]-**OD2**/**OD3** (0.5 μM) was treated with the indicated equivalents of the self-adduct **OD1**-QM2 in NaCl (150 mM), $MgCl_2$ (2.5 mM) and MES (20 mM pH 5) for 5 days at ambient temperature before analysis by denaturing polyacrylamide (20%) gel electrophoresis. (B) The yield of alkylation (%) was determined by phosphoimagery and represents the fraction of total signal per lane. This was compared to **OD1**-QM2 equivalents used in reaction. Selected conditions were tested in a second trial to measure the reproducibility. The error bars of these data represent the range of alkylation yields.
